# Therapeutic target discovery using Boolean network attractors: improvements of kali

**DOI:** 10.1098/rsos.171852

**Published:** 2018-02-14

**Authors:** Arnaud Poret, Carito Guziolowski

**Affiliations:** Laboratoire des Sciences du Numérique de Nantes, Nantes, France

**Keywords:** therapeutic target, biological network, Boolean network, bladder cancer, attractor, drug discovery

## Abstract

In a previous article, an algorithm for identifying therapeutic targets in Boolean networks modelling pathological mechanisms was introduced. In the present article, the improvements made on this algorithm, named kali, are described. These improvements are (i) the possibility to work on asynchronous Boolean networks, (ii) a finer assessment of therapeutic targets and (iii) the possibility to use multivalued logic. kali assumes that the attractors of a dynamical system, such as a Boolean network, are associated with the phenotypes of the modelled biological system. Given a logic-based model of pathological mechanisms, kali searches for therapeutic targets able to reduce the reachability of the attractors associated with pathological phenotypes, thus reducing their likeliness. kali is illustrated on an example network and used on a biological case study. The case study is a published logic-based model of bladder tumorigenesis from which kali returns consistent results. However, like any computational tool, kali can predict but cannot replace human expertise: it is a supporting tool for coping with the complexity of biological systems in the field of drug discovery.

## 1. Introduction

In a previous article, an algorithm for *in silico* therapeutic target discovery was presented in its first version [1]. In the present article, the improvements made on this algorithm, named kali, are described. The complete background was introduced in the previous article, some important concepts of which are recalled in electronic supplementary material, appendix S1.

kali still belongs to the logic-based modelling formalism [2–4], mainly Boolean networks [5,6], and keeps its original goal: searching for therapeutic interventions aimed at healing a supplied pathologically disturbed biological network. Such a network is intended to model the biological mechanisms of a studied disease, on which kali operates. Therapeutic interventions are combinations of targets, these combinations being named bullets. Targets are network components, such as enzymes or transcription factors, and can be subjected to inhibition or activation. This is what bullets specify: which targets and which actions to apply on them.

The pivotal assumption on which kali is based postulates that the attractors of a dynamical system, such as a Boolean network, are associated with the phenotypes of the modelled biological system. In other words, attractors model phenotypes [7]. This assumption was successfully applied in several works [8–14] and makes sense because the steady states of a dynamical system, the attractors, should mirror the steady states of the modelled biological system, the phenotypes.

In the meantime, various works using logical modelling with application in therapeutic innovation were published. An example is the work of Hyunho Chu and co-workers [15]. They built a molecular interaction network involved in colorectal tumorigenesis and studied its dynamics, particularly its attractors and their basins, with stochastic Boolean modelling. They highlighted what they termed the flickering, that is the displacement of the system from one basin to another one due to stochastic noise. They suggested that the flickering is involved in pushing the system from a physiological state to a pathological one during colorectal tumorigenesis.

Concerning kali, three improvements were made: (i) adding the possibility to work with asynchronous Boolean networks, (ii) implementing a finer assessment of therapeutic targets and (iii) adding the possibility to use multivalued logic. The technical features resulting from these improvements are illustrated on a simple example network while their biological significance is assessed in a case study, namely a published logic-based model of bladder tumorigenesis [16].

### 1.1. Handling asynchronous updating

To compute the behaviour of a discrete dynamical system, such as a Boolean network, its variables have to be iteratively updated. These iterative updates can be made synchronously or not [17]. If all the variables are simultaneously updated at each iteration then the network is synchronous, otherwise it is asynchronous. Compared to an asynchronous updating, the synchronous one is easier to compute. However, when the dynamics of a biological network is computed synchronously, it is assumed that all its components evolve simultaneously, an assumption which can be inappropriate according to what is modelled.

The asynchronous updating is frequently built so that one randomly selected variable is updated at each iteration. This allows to capture two important features: (i) biological entities do not necessarily evolve simultaneously and (ii) noise due to randomness can affect when biological interactions take place [18–20]. This is particularly true at the molecular scale, such as with signalling pathways, where macromolecular crowding and Brownian motion can impact the firing of biochemical reactions [21].

Therefore, the choice between a synchronous and an asynchronous updating may depend on the model, the computational resources and the acceptability of synchrony. Knowing that the luxury is to have the choice, kali can now use synchronous and asynchronous updating.

### 1.2. Managing basin sizes for therapeutic purpose

Until now, kali requires therapeutic bullets to remove all the attractors associated with pathological phenotypes, here named pathological attractors. This criterion for selecting therapeutic bullets is somewhat drastic. A smoother criterion should enable to consider more targeting strategies and then more possibilities for counteracting diseases. However, it could also unravel less effective therapeutic bullets, but being too demanding potentially leads to no results and the loss of nonetheless interesting findings.

The therapeutic potential of bullets could be assessed by estimating their ability at reducing the size of the pathological basins, namely the basins of pathological attractors. This criterion is more permissive since therapeutic bullets no longer have to necessarily remove the pathological attractors. Reducing the size of a pathological basin renders the corresponding pathological attractor less reachable and then the associated pathological phenotype less likely. This new criterion includes the previous one: removing an attractor means reducing its basin to the empty set. Consequently, therapeutic bullets obtainable with the previous criterion are still obtainable.

### 1.3. Extending to multivalued logic

One of the main limitations of Boolean models is that their variables can take only two values, which can be too simplistic in some cases. Depending on what is modelled, such as activity level of enzymes or abundance of gene products, considering more than two levels can be better. Without leaving the logic-based modelling formalism, one solution is to extend Boolean logic to multivalued logic [22]. With multivalued logic, a finite number *h* of values in the interval of real numbers [0;1] is used, thus allowing variables to model more than two levels. For example, the level 0.5 can be introduced to model partial activation of enzymes or moderate concentration of gene products.

## 2. Material and methods

### 2.1. Additional definitions

In addition to the background introduced in the previous article [1] and briefly recalled in electronic supplementary material, appendix S1, here are some supplementary definitions:

— *physiological state space*: the state space *S*_physio_ of the physiological variant
— *pathological state space*: the state space *S*_patho_ of the pathological variant
— *testing state space*: the state space *S*_test_ of the pathological variant under the effect of a bullet
— *physiological basin*: the basin *B*_physio,*i*_ of a physiological attractor *a*_physio,*i*_
— *pathological basin*: the basin *B*_patho,*i*_ of a pathological attractor *a*_patho,*i*_
— **n*-bullet*: a bullet made of *n* targets.

### 2.2. Handling asynchronous updating

To incorporate asynchronous updating, the corresponding algorithms coming from BoolNet were implemented into kali. BoolNet is an R [23] package for generation, reconstruction and analysis of Boolean networks [24]. Asynchronous updating is implemented so that one randomly selected variable is updated at each iteration. This random selection is made according to a uniform distribution and implies that the network is no longer deterministic. To do so, given a Boolean network, BoolNet uses the three following functions:

— *AsynchronousAttractorSearch*: this function computes the attractor set of a supplied Boolean network by using the two following functions.
— *ForwardSet*: this function computes the forward reachable set (see below) of a state and considers it as a candidate attractor.
— *ValidateAttractor*: this function checks if a forward reachable set is a terminal strongly connected component (terminal SCC, see below) that is an attractor.

The forward reachable set Fwd_***x***_⊂*S* of a state ***x***∈*S* is the set made of the states reachable from ***x***, including ***x*** itself. A terminal SCC is a set tSCC⊂ *S* made of the forward reachable sets of its states: ∀***x***∈tSCC, Fwd_***x***_⊂tSCC. As a consequence, when a terminal SCC is reached, the system cannot escape it: this is an attractor in the sense of asynchronous Boolean networks [25].

Asynchronous Boolean networks with random updating are not deterministic: their attractors are no longer deterministic sequences of states, namely cycles, but terminal SCCs. To find such an attractor, a long random walk is performed in order to reach an attractor with high probability. This candidate attractor is then validated, or not, by checking if it is a terminal SCC.

### 2.3. Managing basin sizes for therapeutic purpose

To implement the new criterion for selecting therapeutic bullets, kali considers a bullet as therapeutic if it increases the union of the physiological basins $⋃B_{\mathrm{physio},i}$ in the testing state space *S*_test_ without creating de novo attractors. Knowing that an attractor is either physiological or pathological, increasing $⋃B_{\mathrm{physio},i}$ is equivalent to decreasing $⋃B_{\mathrm{patho},i}$.

The goal is to increase the physiological part of the pathological state space, or equivalently to decrease its pathological part. Consequently, a pathologically disturbed biological network receiving such a therapeutic bullet tends to, but not necessarily reaches, an overall physiological behaviour.

However, as with the previous criterion, it does not ensure that all the physiological attractors are preserved. *A fortiori*, it does not ensure that their basin remains unchanged. It means that a therapeutic bullet can also alter the reachability of the physiological attractors. Nevertheless, as with the previous criterion, this is a matter of choice between a therapeutic bullet or no bullet at all.

The therapeutic potential of a bullet is expressed by its gain. It is displayed as follows:

$$x\%\to y\%\;\text{with }x=100\times \frac{|⋃B_{\mathrm{physio},i}|}{|S_{\mathrm{patho}}|}\;\text{and}\;y=100\times \frac{|⋃B_{\mathrm{physio},i}|}{|S_{\mathrm{test}}|},$$

expressed in percentages. Therefore, in order to increase the physiological part of the pathological state space, a therapeutic bullet has to make *y*≥*x*.

Note that *y*=*x* is allowed. In this particular case, it is conceivable that the size of several pathological basins changed while the size of their union did not. In other words, the composition of the pathological part changed while its size did not. It can be therapeutic if, for example, the basin of a weakly pathological attractor increased at the expense of the basin of a heavily pathological attractor.

The increase of the physiological part of the pathological state space can be subjected to a threshold *δ*: *y*≥*x* becomes *y*−*x*≥*δ*. As *x* and *y*, *δ* is expressed in percentages of the state space. This threshold is introduced in order to allow the stringency of kali to be tuned. By the way, using this threshold also decreases the probability to obtain misassessed therapeutic bullets due to round-off errors, or sampling errors when the state space is too big to compute trajectories from each of the possible states.

A therapeutic bullet as defined by the previous criterion, namely which removes all the pathological attractors, makes de facto $⋃B_{\mathrm{physio},i}=100\%$ of *S*_test_. As already mentioned, the previous criterion is included in this new one: therapeutic bullets obtainable with the former are also obtainable with the latter.

It must be pointed out that the current implementation of the method described in this article, namely kali, computes basin sizes by counting the number of initial states leading to a given attractor. If these initial states are a subset of the state space then basin sizes are estimations. Moreover, if an asynchronous updating is used then the system is not deterministic, implying that an initial state can lead to more than one attractor. Consequently, in those cases, basin sizes and therapeutic gains are estimations also subjected to random variations.

In other words, concerning the calculation of basin sizes, the current implementation of kali is more an attractor reachability estimation than a true basin size calculation. Nevertheless, speaking in terms of basins is kept in order to better comply with the underlaying method, independently of its implementation which is subjected to further improvements.

### 2.4. Extending to multivalued logic

Extending to multivalued logic requires suitable operators to be introduced. One solution is to use an implementation of the Boolean operators which also works with multivalued logic, just as the Zadeh operators. These operators are a generalization of the Boolean ones proposed for fuzzy logic by its pioneer Lotfi Zadeh [26]. Their formulation is

$$\begin{array}{rl} x\wedge y & =\min(x,y),\\ x\vee y & =\max(x,y)\\ \;\mathrm{and}\;\neg x & =1-x. \end{array}$$

With an *h*-valued logic, the size of the *n*-dimensional state space is *h*^*n*^, bringing more computational difficulties than with Boolean logic. The same applies to the testable bullets (see below) because there are *h*^*r*^ possible modality arrangements and then (*n*!⋅*h*^*r*^)/(*r*!⋅(*n*−*r*)!) possible bullets, where *r* is the number of targets per bullet.

As introduced in the previous article [1] and recalled in electronic supplementary material, appendix S1, a bullet is a couple (*c*_targ_,*c*_moda_) where *c*_targ_=(targ_1_,…,targ_*r*_) is a combination without repetition of *r* nodes and *c*_moda_=(moda_1_,…,moda_*r*_) is an arrangement with repetition of *r* perturbations, here termed modalities. moda_*i*_ is intended to be applied on targ_*i*_.

To illustrate how kali works with multivalued logic without overloading it, a three-valued logic is used with {0,0.5,1} as domain of value: *x*_*i*_∈{0,0.5,1}. 0 and 1 have the same meaning as with Boolean logic. 0.5 is an intermediate truth degree which can be interpreted as an intermediate level of activity/abundance depending on what the variables refer to. By the way, *S*={0,0.5,1}^*n*^ and moda_*i*_∈{0,0.5,1}.

### 2.5. Example network

**Figure 1.**
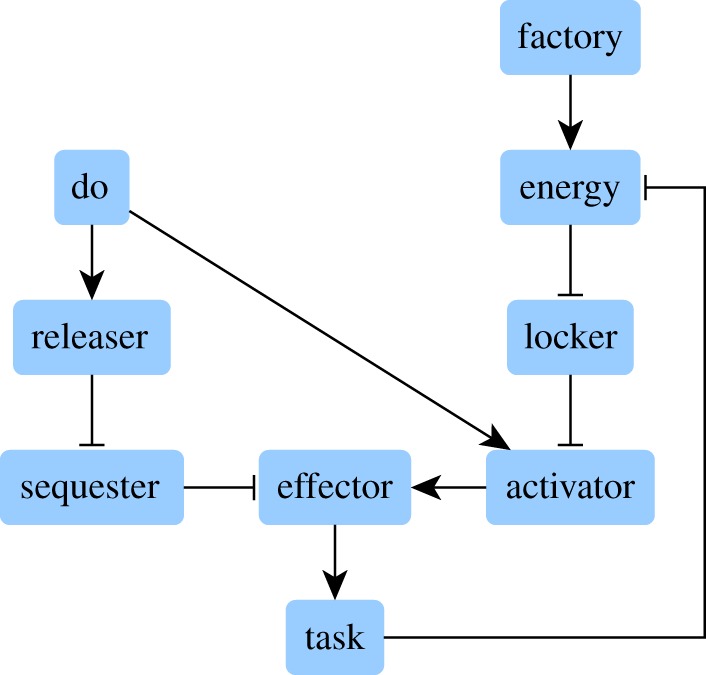
This network, running in a fictive cell, controls the execution of a task according to two inputs: (i) the do instruction, which tells the task to be performed, and (ii) energy supply. The task consumes energy and must be prevented if no energy is available, even if the do instruction is sent. The task is initiated by an effector, which is maintained inactive by a sequester. The do instruction activates a releaser which suppresses the sequestering activity of the sequester, thus releasing the effector. However, to initiate the task and in addition to be released, the effector has also to be activated by an activator. When released and activated, the effector initiates the task. To ensure that the task is performed only if energy is available, a locker maintains the activator in an inactive state if there is no energy, even if the do instruction is sent. With regard to the factory, it supplies energy.

To conveniently illustrate the technical features resulting from the improvements made on kali, a simple and fictive example network is used. A biological case study is then proposed to address a concrete case, namely a published logic-based model of bladder tumorigenesis [16]. The example network is depicted in figure 1.

Among the three improvements made on kali, only the asynchronous updating and the management of basin sizes are illustrated. Multivalued logic is a straightforward extension of the Boolean case and is illustrated in electronic supplementary material, appendix S2. Below are the Boolean equations encoding the example network, also available in text format in the electronic supplementary material, example_equations.txt:

$$\begin{array}{rl} \mathrm{do} & =\mathrm{do}\\ \mathrm{factory} & =\mathrm{factory}\\ \mathrm{energy} & =\mathrm{factory}\vee (\mathrm{energy}\wedge \neg \mathrm{task})\\ \mathrm{locker} & =\neg \mathrm{energy}\\ \mathrm{releaser} & =\mathrm{do}\\ \mathrm{sequester} & =\neg \mathrm{releaser}\\ \mathrm{activator} & =\mathrm{do}\wedge \neg \mathrm{locker}\\ \mathrm{effector} & =\mathrm{activator}\wedge \neg \mathrm{sequester}\\ \mathrm{task} & =\mathrm{effector}. \end{array}$$

The do instruction and the factory are the two inputs: they are constant and thus equal to themselves. The equation of energy tells us that energy is present if the factory is active, even when the task is running: the factory has a sufficient production capacity. However, if the factory is not active then energy disappears as soon as the task is initiated. With regard to the activator and the effector, their equations tell us that their respective inhibitor takes precedence: whatever the state of the other nodes, if the inhibitor is active then the target is not.

The physiological variant ***f***_physio_ is the network as is. The pathological variant ***f***_patho_ is the network plus a constitutive inactivation of the locker: the execution of the task no longer considers if energy is available. Consequently, *f*_locker_ becomes locker=0 in ***f***_patho_.

### 2.6. Case study: bladder tumorigenesis

This case study consists in running kali on a logic-based model of bladder tumorigenesis published by Elisabeth Remy and co-workers [16]. Elisabeth Remy and co-workers have built an influence network linking three extracellular input signals and one intracellular input event to three cellular output phenotypes.

The three extracellular input signals are growth stimulations, represented by the *EGFRstimulus* and *FGFR3stimulus* parameters, and growth inhibitions, mainly modelling TGF-*β* effects and represented by the *GrowthInhibitors* parameter. The intracellular input event is DNA damage, represented by the *DNAdamage* parameter. The three cellular output phenotypes are proliferation, growth arrest and apoptosis. The model integrates downstream effectors of growth factor receptors such as Ras and PI3K, growth inhibitors such as p14ARF and p16INK4a, and regulators of the cell cycle such as cyclinD1, E2F3 and pRb.

Some variables are ternary: they can take three possible values in order to account for different effects depending on the activation level. These three possible values are 0 and 1 as in the Boolean case, plus the additional level 2. As in the model implementation performed by Elisabeth Remy and co-workers, these ternary variables are translated into pairs of Boolean variables: one Boolean variable per activation level, namely level 1 and level 2.

For example and according to the model, in its normal expression level (level 1, *E*2*F*1=1) the transcription factor E2F1 stimulates the expression of genes supporting the cell cycle. However, when over-expressed (level 2, *E*2*F*1=2) E2F1 stimulates the expression of genes supporting apoptosis. Consequently, this ternary variable is translated into the pair of Boolean variables *E*2*F*1_*lvl*1_ and *E*2*F*1_*lvl*2_:

$$\begin{array}{rl} E2F1 & =1\;\Leftrightarrow \;E2F1_{lvl1}=1\\ \;\mathrm{and}\;E2F1 & =2\;\Leftrightarrow \;E2F1_{lvl2}=1. \end{array}$$

The variable modelling the output phenotype *Apoptosis* is one of these ternary variables. The goal of Elisabeth Remy and co-workers was to relate apoptosis to its trigger: p53-dependent apoptosis (*Apoptosis*_*lvl*1_) and E2F1-dependent apoptosis (*Apoptosis*_*lvl*2_). However, in this case study, only the cell fate matters. These two trigger-dependent apoptosis are therefore merged into one equation:

$$\mathrm{Apoptosis}={\mathrm{Apoptosis}}_{lvl1}\vee {\mathrm{Apoptosis}}_{lvl2}.$$

As the four inputs of the model are parameters, their respective values are directly injected into the concerned equations so that no equations are dedicated to them, thus reducing computational requirements. Again to reduce computational requirements and knowing that the three output phenotypes are readouts not influencing other variables, their corresponding equations are put out of the model and evaluated from the returned attractors once the run has terminated:

$$\begin{array}{rl} \mathrm{Proliferation} & =\mathrm{Cyclin}E1\vee \mathrm{Cyclin}A\\ \mathrm{GrowthArrest} & =p21CIP\vee RB1\vee RBL2\\ \;\mathrm{Apoptosis} & =TP53\vee E2F1_{lvl2}. \end{array}$$

**Figure 2.**
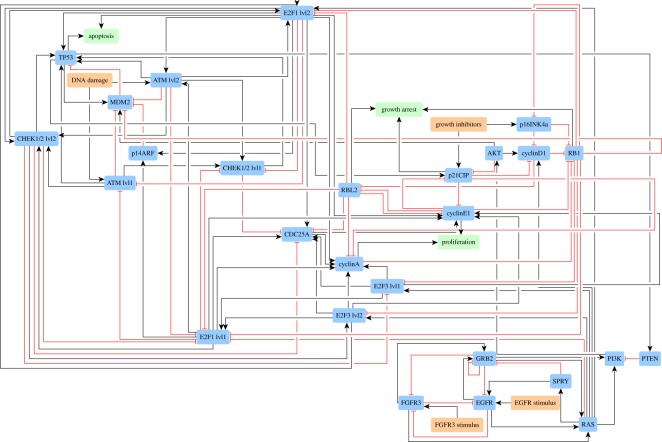
A network-based representation of the case study used to assess kali on a concrete case. As explained in the text, it is derived from a published logic-based model of bladder tumorigenesis [16]. Nodes represent Boolean variables while edges indicate positive (black) and negative (red) influences. The input signals/events growth stimulations, growth inhibitions and DNA damage are in red while the output phenotypes proliferation, growth arrest and apoptosis are in green.

Altogether, the above-described adaptations made on the model of bladder tumorigenesis published by Elisabeth Remy and co-workers give a case study of 27 Boolean equations. These equations are listed in electronic supplementary material, appendix S4, also available in text format in the electronic supplementary material, bladder_equations.txt. A network-based representation is shown in figure 2.

The physiological variant ***f***_physio_ is the model as is. The pathological variant ***f***_patho_ is the model plus a deletion of the tumour suppressor gene CDKN2A, as observed in bladder cancers [27,28]. Note that the CDKN2A gene encodes two growth inhibitors: p14ARF and p16INK4a. Consequently, the equations modelling these two variables become *p*14*ARF*=0 and *p*16*INK*4*a*=0 in ***f***_patho_.

### 2.7. Implementation, code availability, licence

kali is implemented in Go [29] and tested with Go version go1.9.2 linux/amd64 under Arch Linux [30]. kali is licensed under the GNU General Public License [31] and freely available on GitHub at https://github.com/arnaudporet/kali. The core of kali in pseudocode can be found in electronic supplementary material, appendix S3.

## 3. Results

### 3.1. Example network

#### 3.1.1. Attractor sets

The example network is computed asynchronously over the whole state space, namely 512 possible initial states, using Boolean logic. As explained in the Material and methods section, the asynchronous attractor search uses long random walks to reach candidate attractors with high probability, and then checks if they are indeed true attractors. Owing to the small size of the example network, the length $\max_{k}$ of these random walks is set to 1000 steps. With larger state spaces, random walks should be longer to reach candidate attractors with high probability.

The resulting attractors can be studied along four variables: the do instruction, the factory, the locker and the task. It is possible for energy to be present without a running factory in the initial conditions. In this case, if the do instruction is sent then energy is consumed by the task but not remade by the factory. With the physiological variant, the locker is expected to stop the task. However, with the pathological variant where the locker is disabled, an abnormal behaviour is expected. Below are the computed attractors:

— *A*_physio_:
  **Table:** 

attractor	basin (% of *S*_physio_)	do	factory	energy	locker	task
*a*_physio1_	17.8%	0	0	0	1	0
*a*_physio2_	7.2%	0	0	1	0	0
*a*_physio3_	25%	0	1	1	0	0
*a*_physio4_	25%	1	0	0	1	0
*a*_physio5_	25%	1	1	1	0	1
— *A*_patho_:
  **Table:** 

attractor	basin (% of *S*_patho_)	do	factory	energy	locker	task
*a*_patho1_	18.4%	0	0	0	0	0
*a*_physio2_	6.6%	0	0	1	0	0
*a*_physio3_	25%	0	1	1	0	0
*a*_patho2_	25%	1	0	0	0	1
*a*_physio5_	25%	1	1	1	0	1

With the physiological variant, the behaviour is as expected: the task runs only if the do instruction is sent and only if the factory can remake the consumed energy. With the pathological variant, two pathological phenotypes represented by *a*_patho1_ and *a*_patho2_ appear. *a*_patho1_ is pathological because the locker is inactive while there is no available energy. However, it is weakly pathological because the do instruction is not sent: there is no task to stop, an operational locker is not mandatory.

By contrast, *a*_patho2_ is heavily pathological because an operational locker is required to stop the task in the absence of energy supply. In the fictive cell bearing this example network, *a*_patho2_ could drain all its energy content, thus bringing it to thermodynamical death. Moreover, *a*_patho2_ should not be neglected because its basin occupies 25% of the pathological state space.

#### 3.1.2. Therapeutic bullets

Bullets are assessed for their therapeutic potential on the pathological variant ***f***_patho_ according to the new criterion: decreasing the size of the pathological basins *B*_patho,*i*_. All the bullets made of one to two targets are tested with a threshold of 5%.

Choosing a threshold can appear somewhat arbitrary. It tells us that if the physiological part $⋃B_{\mathrm{physio},i}$ in the pathological state space *S*_patho_ occupies *x*% of it, then to be therapeutic a bullet has to bring this value above (*x*+5)% in the testing state space *S*_test_. Therefore, the increases below this threshold are considered not significant by kali. Even the choice of using a threshold can be arbitrary, as discussed in the Material and methods section.

Knowing that $⋃B_{\mathrm{physio},i}=56.6\%$ of *S*_patho_, with a threshold of 5% the 1,2-bullets have to make $⋃B_{\mathrm{physio},i}\geq (56.6+5)\%=61.6\%$ of *S*_test_ to be considered therapeutic. Below are the returned therapeutic bullets:

— 1-therapeutic bullets:
  **Table:** 

bullet	gain			*B*_physio1_	*B*_physio2_	*B*_physio3_	*B*_physio4_	*B*_physio5_	*B*_patho1_	*B*_patho2_
do[0]	56.6%	→	64.4%	0%	14.4%	50%	0%	0%	35.5%	0%
factory[1]	56.6%	→	100%	0%	0%	50%	0%	50%	0%	0%
— 2-therapeutic bullets:
  **Table:** 

bullet		gain			*B*_physio1_	*B*_physio2_	*B*_physio3_	*B*_physio4_	*B*_physio5_	*B*_patho1_	*B*_patho2_
do[0]	factory[1]	56.6%	→	100%	0%	0%	100%	0%	0%	0%	0%
do[1]	factory[1]	56.6%	→	100%	0%	0%	0%	0%	100%	0%	0%
do[0]	energy[1]	56.6%	→	100%	0%	50%	50%	0%	0%	0%	0%
do[0]	locker[0]	56.6%	→	64.1%	0%	14.1%	50%	0%	0%	35.9%	0%
do[0]	releaser[0]	56.6%	→	62.9%	0%	12.9%	50%	0%	0%	37.1%	0%
do[0]	sequester[1]	56.6%	→	62.5%	0%	12.5%	50%	0%	0%	37.5%	0%
do[0]	activator[0]	56.6%	→	64.8%	0%	14.8%	50%	0%	0%	35.2%	0%
do[0]	effector[0]	56.6%	→	67.8%	0%	17.8%	50%	0%	0%	32.2%	0%
do[0]	task[0]	56.6%	→	73.2%	0%	23.2%	50%	0%	0%	26.8%	0%
factory[1]	energy[1]	56.6%	→	100%	0%	0%	50%	0%	50%	0%	0%
factory[1]	locker[0]	56.6%	→	100%	0%	0%	50%	0%	50%	0%	0%

where *x*[*y*] means that the variable *x* has to be set to the value *y*. For example, the therapeutic bullet do[0] factory[1] suggests to abolish the do instruction while maintaining the factory active.

All the returned therapeutic bullets not removing all the pathological attractors exhibit the ability to suppress the basin of *a*_patho2_ while increasing the one of *a*_patho1_. Certainly, removing all the pathological attractors should be better, but knowing the *a*_patho2_ is more pathological than *a*_patho1_, such therapeutic bullets can nevertheless be interesting. With the previous criterion, namely removing all the pathological attractors, these therapeutic bullets are not obtainable, thus highlighting fewer therapeutic strategies.

Some of the found therapeutic bullets enable physiological attractors required by the pathological variant to react properly to the do instruction. For example, the therapeutic bullet factory[1] enables *a*_physio3_ and *a*_physio5_, corresponding respectively to ‘no do, no task’ and ‘do the task, energy supply’. However, the remainder of the therapeutic bullets, such as do[0] releaser[0] or do[1] factory[1], either disable or force the do instruction, thus either suppressing or forcing the task. A network unable to perform the task or, at the opposite, permanently doing it may not be therapeutically interesting, even if energy is supplied.

None of the found therapeutic bullets suggest to reverse the constitutive inactivation of the locker. This highlights that applying the opposite action of the pathological disturbance is not necessarily a therapeutic solution, which can appear counterintuitive. This is because biological entities subjected to pathological disturbances belong to complex networks exhibiting behaviours which cannot be mentally computed [32,33]. In such a context, computational tools and their growing computing capabilities can help owing to their integrative power [34–38].

Also, none of the found therapeutic bullets allow the recovery of all the physiological attractors: there are no golden bullets. In a general manner, the components of biological networks should be able to take several states, such as enzymes which should be active when suitable. Consequently, healing a pathologically disturbed biological network by maintaining some of its components in a particular state should not allow the recovery of a complete and healthy behaviour. This is a limitation of the method implemented in kali.

This limitation is common in biomedicine while not necessarily being an issue. For example, statins are well-known lipid-lowering drugs widely used in cardiovascular diseases with proven benefits [39,40]. They inhibit an enzyme, the HMG-CoA reductase, and they do it constantly, just as the targets are modulated in the therapeutic bullets returned by kali. The HMG-CoA reductase belongs to a complex metabolic network and maintaining it in an inhibited state should not allow this network to run properly, maybe causing some adverse effects. Nevertheless, such as with all drugs, this is a matter of benefit–risk ratio.

All of this indicates that there are no perfect strategies for counteracting diseases and that computational tools, such as kali, can help scientists but cannot replace their expertise. Human expertise is mandatory to assess the returned predictions according to a concrete setting, and ultimately to take decisions.

### 3.2. Case study: bladder tumorigenesis

#### 3.2.1. Attractor sets

The case study is computed asynchronously using Boolean logic. The state space being quite big with 134 217 728 possible states, to compute an attractor set kali performs random walks starting from 1000 randomly selected initial states. A bigger state space also requires these random walks to be longer in order to reach candidate attractors with high probability. The length max_*k*_ of the random walks is then increased to 10 000 steps.

The four input parameters of the model are tuned to simulate a biological situation where undamaged cells receive both growth-stimulating and growth-inhibiting signals from their environment:

$$\begin{array}{rl} EGFRstimulus & =1\\ FGFR3stimulus & =1\\ \mathrm{GrowthInhibitors} & =1\\ \mathrm{DNAdamage} & =0. \end{array}$$

This input configuration aims at predicting the possible responses of the model to opposite growth instructions. In a cancerous setting, it is desirable that the growth-inhibiting signal takes precedence over the stimulating one. With the pathological variant where the two growth inhibitors p14ARF and p16INK4a are absent, this desired precedence might be compromised in favour of tumorigenesis, thus correlating with the observed CDKN2A gene deletion in bladder cancers [27,28].

The phenotypes associated with the returned attractors are evaluated using their respective equation once the run has terminated, as explained in the Material and methods section. Below are the computed attractors together with their phenotypes and basins, expressed in percentages of the corresponding state space:

**Table:** 

	*A*_physio_			*A*_patho_	
name	*a*_physio1_	*a*_physio2_	*a*_physio3_	*a*_physio1_	*a*_patho1_
basin	10.7%	74.5%	14.8%	65.4%	34.6%
phenotype	GA	GA	P	GA	P
*AKT*	0	0	0	0	0
*ATM*_*lvl*1_	0	0	0	0	0
*ATM*_*lvl*2_	0	0	0	0	0
*CDC*25*A*	0	0	1	0	1
*CHEK*1/2_*lvl*1_	0	0	0	0	0
*CHEK*1/2_*lvl*2_	0	0	0	0	0
*CyclinA*	0	0	1	0	1
*CyclinD*1	0	0	0	0	1
*CyclinE*1	0	0	1	0	1
*E*2*F*1_*lvl*1_	0	0	1	0	1
*E*2*F*1_*lvl*2_	0	0	0	0	0
*E*2*F*3_*lvl*1_	0	1	1	0	1
*E*2*F*3_*lvl*2_	0	0	0	0	0
*EGFR*	0	0	0	0	0
*FGFR*3	1	1	1	1	1
*GRB*2	0	0	0	0	0
*MDM*2	0	0	0	0	0
*p*14*ARF*	0	0	1	0	0
*p*16*INK*4*a*	0	1	1	0	0
*p*21*CIP*	1	1	0	1	0
*PI*3*K*	0	0	0	0	0
*PTEN*	0	0	0	0	0
*RAS*	1	1	1	1	1
*RB*1	1	0	0	1	0
*RBL*2	1	1	0	1	0
*SPRY*	1	1	1	1	1
*TP*53	0	0	0	0	0

where GA means growth arrest and P means proliferation.

The physiological variant is able to exhibit the two possible responses according to the input configuration: proliferation, represented by *a*_physio3_, and growth arrest, represented by *a*_physio1_ and *a*_physio2_. Growth arrest occupies 85.2% of the physiological state space, suggesting that normal cells are more likely to comply with growth-inhibiting signals than with stimulating ones.

With the pathological variant modelling cells whose two growth inhibitors p14ARF and p16INK4a are lost, the two possible responses are still present with again growth arrest being more likely than proliferation. Even if *a*_physio2_ disappears, growth arrest is still possible with *a*_physio1_ whose basin increases from 10.7% in *S*_physio_ to 65.4% in *S*_patho_. The proliferating phenotype is also still possible but through the pathological attractor *a*_patho1_ which, in a way, replaces the physiological attractor *a*_physio3_.

However, the global tendency towards growth arrest significantly decreases: proliferation is more than twice as likely in the pathological variant than in the physiological one with a shift from 14.8% in *S*_physio_ to 34.6% in *S*_patho_. Therefore, such pathological cells might be less responsive to growth-inhibiting signals and more apt at proliferating, which is a major concern in tumorigenesis and consistent with the loss of two growth inhibitors.

To ensure that browsing the state space by performing 1000 random walks of 10 000 steps is sufficient to find all the attractors while estimating their basin with little variability, the physiological and pathological attractor sets were computed 100 times each:

**Table:** 

set	attractor	basin (% of *S*)
*A*_physio_	*a*_physio1_	10.518±0.833
	*a*_physio2_	73.462±1.24
	*a*_physio3_	16.02±1.091
*A*_patho_	*a*_physio1_	65.037±1.687
	*a*_patho1_	34.963±1.687

These results indicate that, in this case study, browsing the state space by performing 1000 random walks of 10 000 steps is robust enough to obtain reproducible results. Indeed, at each time, the same attractors are found: no attractor is missed. Moreover, the means of the basin estimations exhibit low standard deviations: basin estimations are subjected to variability but are nonetheless reliable.

#### 3.2.2. Therapeutic bullets

As in the example network, bullets are assessed for their therapeutic potential on the pathological variant ***f***_patho_ according to the new criterion: increasing the physiological part $⋃B_{\mathrm{physio},i}$ in the testing state space *S*_test_ with a threshold of 5%. It means that therapeutic bullets have to push $⋃B_{\mathrm{physio},i}$ from 65.4% in *S*_patho_ to at least 65.4+5=70.4% in *S*_test_.

In this case study belonging to a cancerous setting, it is desirable that therapeutic bullets also promote growth arrest in order to slow down tumorigenesis. In terms of basins and attractors, it means that interesting therapeutic bullets should decrease *B*_patho1_, avoid *a*_physio3_, increase *B*_physio1_ and reintroduce *a*_physio2_. Such therapeutic bullets could be qualified as anti-proliferative.

All the 1458 bullets made of one to two targets are tested. Among them, kali finds nine 1-therapeutic bullets and 174 2-therapeutic bullets listed in the electronic supplementary material, bladder_B_therap_1.txt and bladder_B_therap_2.txt respectively. In addition to increasing the physiological part, all the returned therapeutic bullets are anti-proliferative. Indeed, all of them do not reintroduce *a*_physio3_ and decrease *B*_patho1_, thus promoting growth arrest through *a*_physio1_ and/or *a*_physio2_.

For example, the two following 1-therapeutic bullets increase *B*_physio1_ while decreasing *B*_patho1_, thus exhibiting an anti-proliferative effect as expected when targeting the well-known growth-promoting PI3K/Akt pathway [41]:

**Table:** 

bullet	gain			*B*_physio1_	*B*_physio2_	*B*_physio3_	*B*_patho1_
*AKT*[0]	65.4%	→	89.3%	89.3%	0%	0%	10.7%
*PI*3*K*[0]	65.4%	→	86%	86%	0%	0%	14%

Below is another interesting 1-therapeutic bullet predicting that inhibiting CDC25A is anti-proliferative:

**Table:** 

bullet	gain			*B*_physio1_	*B*_physio2_	*B*_physio3_	*B*_patho1_
*CDC*25*A*[0]	65.4%	→	100%	100%	0%	0%	0%

This therapeutic bullet is able to definitively suppress proliferation by making *B*_physio1_=100% of *S*_test_. It makes sense because the tyrosine phosphatase CDC25A can activate several cyclin-dependent kinases (CDKs) which, with their cyclin partners, promote cell cycle and then growth [42]. This prediction correlates with biological knowledge about CDC25A inhibitors as potential anti-cancer agents [43]. For example, it is demonstrated that inhibiting CDC25A suppresses the growth of hepatocellular carcinoma cells [44,45]. Moreover, a recent work was specially dedicated to the synthesis of anti-cancer agents inhibiting the CDC25A/B phosphatases [46].

This highlights that dry-lab predictions consistent with factual evidence coming from wet-lab experiments are obtainable through kali, provided that the underlying model is consistent too. Note that this does not imply that all the predictions are correct: needless to say, biological interpretation by experts is still mandatory.

The 2-therapeutic bullets also bring some interesting predictions. For example, they indicate that sprouty (SPRY) could be a therapeutic target but only in combination with another one: there are no 1-therapeutic bullets containing it. Sprouty negatively regulates mitogen-activated protein kinase (MAPK) signalling pathways downstream of growth factor receptors and is down-regulated in many cancers [47]. Consequently, stimulating sprouty should be anti-proliferative and this is what suggests the two following therapeutic bullets, even if the gain is relatively minor:

**Table:** 

bullet		gain			*B*_physio1_	*B*_physio2_	*B*_physio3_
					*B*_patho1_		
*E*2*F*3_*lvl*2_[0]	*SPRY* [1]	65.4%	→	70.5%	70.5%	0%	0%
					29.5%		
*MDM*2[0]	*SPRY* [1]	65.4%	→	71.7%	71.7%	0%	0%
					28.3%		

These two therapeutic bullets indicate that stimulating sprouty should be done along with an inhibition of MDM2 or E2F3. As with CDC25A, this prediction correlates with biological knowledge: MDM2 is a major inhibitor of the well-known tumour suppressor p53 [48] while E2F3 is a required transcription factor for the cell cycle [49]. However, this concerns only the level 2 of E2F3, meaning that only its over-expression should be prevented. In other words, this is not an inhibition of E2F3 but rather the prevention of its over-expression, if any.

In the returned therapeutic bullets, there are also intriguing results such as the following one:

**Table:** 

bullet	gain			*B*_physio1_	*B*_physio2_	*B*_physio3_	*B*_patho1_
*FGFR*3[1]	65.4%	→	74.1%	74.1%	0%	0%	25.9%

This therapeutic bullet moderately increases *B*_physio1_ at the expense of *B*_patho1_, therefore promoting growth arrest. However, FGFR3 is a growth factor receptor and is frequently subjected to activating mutations in low-grade bladder cancers [50]. Therefore, stimulating FGFR3 should promote proliferation, not growth arrest. However, Elisabeth Remy and co-workers have implemented a negative cross-talk from FGFR3 to the growth factor receptor EGFR in their model. This negative cross-talk may explain why stimulating FGFR3 is predicted to be anti-proliferative.

Indeed, *EGFR*[0] is one of the returned therapeutic bullets and represents a direct inhibition of EGFR, a well-studied target in cancer therapies [51,52]. Consequently and according to the model, *FGFR*3[1] can be interpreted as an indirect inhibition of EGFR, especially because these two therapeutic bullets have almost identical effects in magnitude:

**Table:** 

bullet	gain			*B*_physio1_	*B*_physio2_	*B*_physio3_	*B*_patho1_
*EGFR*[0]	65.4%	→	75.4%	75.4%	0%	0%	24.6%

Finally, it should be noted that the three following bullets are not predicted therapeutic by kali: *p*14*ARF*[1], *p*16*INK*4*a*[1] and *p*14*ARF*[1] *p*16*INK*4*a*[1]. As with the example network, this suggests that applying the opposite action of the pathological disturbance is not necessarily a therapeutic solution. Moreover, and again as with the example network, none of the found therapeutic bullets allow the recovery of all the physiological attractors: golden bullets seem to be as idealistic as golden pills.

### 3.3. Computation times

The results presented in this article were obtained on a laptop with 16GB of RAM and an Intel Core i7-6600U processor. There are two kali parameters strongly influencing computation times. These two parameters control the attractor search and are:

— $\max_{S}$: the maximum number of initial states to use when computing an attractor set
— $\max_{k}$: the length of the random walks performed to reach candidate attractors.

The asynchronous attractor search consists in performing $\max_{S}$ random walks of $\max_{k}$ steps. Knowing that such a search is performed for computing an attractor set and that one attractor set is computed per tested bullet, the computation time can greatly increase with $\max_{S}$ and/or $\max_{k}$. By the way, computation times also increase with *n*_targ_, $\max_{\mathrm{targ}}$ and $\max_{\mathrm{moda}}$, three kali parameters controlling how much bullets are tested:

— *n*_targ_: the number of targets per bullet
— $\max_{\mathrm{targ}}$: the maximum number of target combinations to test
— $\max_{\mathrm{moda}}$: the maximum number of modality arrangements to test.

The used logic can also increase computation times because the size of the state space is *h*^*n*^, where *n* is the number of nodes in the network and *h* is the number of possible values for the variables. For example, *h*=2 with Boolean logic and *h*=3 with three-valued logic. *h* can also increase the number of testable bullets, and then computation times, because there are (*n*!⋅*h*^*n*_targ_^)/(*n*_targ_!⋅(*n*−*n*_targ_)!) possible bullets.

Below are the computation times of the runs performed for this article:

**Table:** 

	example network (Boolean)	example network (three-valued)	case study (Boolean)
max_*S*_	512 (all)	1000	1000
max_*k*_	1000	1000	10 000
1-bullets	18 (all)	27 (all)	54 (all)
2-bullets	144 (all)	324 (all)	1404 (all)
*A*_physio_	130 ms	187 ms	6s 89 ms
*A*_patho_	109 ms	218 ms	6s 55 ms
*B*_therap_ (*n*_targ_=1)	2s 510 ms	6s 775 ms	5 m 57 s 950 ms
*B*_therap_ (*n*_targ_=2)	19s 133 ms	1 m 23 s 526 ms	2 h43 m 36 s 709 ms

## 4. Conclusion

kali can now work on both synchronous and asynchronous Boolean networks. This is probably the most required improvement because asynchronous updating is frequently used in the scientific community and might be more realistic than synchrony, as discussed in the Introduction section. Consequently, a computational tool aimed at working on models built by the scientific community, such as kali, has to handle this updating scheme.

Also note that there is more than one asynchronous updating scheme. The one implemented in kali is the most popular and is named general asynchronous updating: one randomly selected variable is updated at each iteration. However, other asynchronous updating methods exist. For example, with random-order updating, all the variables are updated at each iteration along a randomly selected order. Implementing various asynchronous updating schemes in kali could be a required future improvement.

kali now uses a new criterion for assessing therapeutic bullets. This new criterion brings a wider range of targeting strategies intended to push pathological behaviours towards physiological ones. It is based on a more permissive assumption stating that reducing the reachability of pathological attractors is therapeutic.

For an *in silico* tool such as kali, being a little bit more permissive can be important because the findings obtained by simulations have to outlive the bottleneck separating predictions and reality. With a too strict assessment of therapeutic bullets, the risk of highlighting too few candidate targets or to miss some interesting ones can be high. Moreover, predicted does not necessarily mean true: a prediction of apparently poor interest can reveal itself to be of great interest, and vice versa.

This new criterion also brings a finer assessment of therapeutic bullets because all the possible increases of $⋃B_{\mathrm{physio},i}$ in *S*_test_ are considered. With the previous criterion, there was only one therapeutic potential: $⋃B_{\mathrm{physio},i}=100\%$ of *S*_test_, thus reducing the assessment of bullets to therapeutic or not. Things are not so dichotomous but rather nuanced: the assessment of therapeutic bullets should be nuanced too.

kali can now work with multivalued logic. Allowing variables to take an arbitrary finite number of values should enable to more accurately model biological processes and produce more fine-tuned therapeutic bullets. However, this accuracy and fine-tuning are at the cost of an increased computational requirement. Indeed, the size of the state space depends on the size of the model and the logic used.

Consequently, the size of the model and the used logic should be balanced: the smaller the model is, the more variables should be finely valued. For example, for an accurate therapeutic investigation, the model should only contain the essential and specific pieces of the studied pathological mechanisms modelled by a finely valued logic. On the other hand, for a broad therapeutic investigation, a more exhaustive model can be used but modelled by a coarse-grained logic.

Note that the ultimate multivalued logic is the infinitely valued one, which is fuzzy logic [53]. With fuzzy logic, the whole interval of real numbers [0;1] is used to valuate variables, which might bring the best accuracy for the qualitative modelling formalism [54–56]. However, using such a continuous logic implies to leave the relatively convenient discrete paradigm to enter the continuous one where, for example, the state space is infinite.

kali also demonstrates that it is able to predict therapeutic bullets consistent with the underlying model, with biological knowledge and with experimental evidences. For example, in the bladder tumorigenesis case study, kali returned therapeutic bullets inhibiting the PI3K/Akt pathway or the CDC25A tyrosine phosphatase, two documented targets in cancer therapies.

Even the surprising *FGFR*3[1] therapeutic bullet, which suggest to stimulate a growth factor receptor for promoting growth arrest, is consistent with the underlying model. Indeed, according to this model, it appears that *FGFR*3[1] is founded in a negative cross-talk from FGFR3 to EGFR, thus indirectly inhibiting the growth factor receptor EGFR, which is also a documented target in cancer therapies.

Two additional improvements are envisaged for kali. The first one is to allow therapeutic bullets to create new attractors, namely de novo attractors. It is conceivable that a bullet can greatly decrease pathological basins while creating a new attractor not belonging to the physiological variant nor to the pathological one. Such a de novo attractor is currently tagged by kali as not physiological and then pathological, thus rejecting the concerned bullet. However, if a de novo attractor is weakly pathological and induced by a bullet greatly decreasing the basin of other and heavier pathological attractors, such a case should be retained.

The second envisaged improvement is to allow partial matching when checking if an attractor is associated with a physiological phenotype by comparing it to the physiological attractors. Currently, an attractor which does not match a physiological attractor is considered pathological. However, it is conceivable that some variables not exhibiting a physiological behaviour in an attractor do not pathologically impact its associated phenotype. To allow such a case to be considered, some variables within attractors should be allowed to not be matched when assessing the associated phenotype.

This suggests the concept of decisive variables, namely variables whose behaviour in the attractors is sufficient to biologically interpret the associated phenotypes. Elisabeth Remy and co-workers have already implemented this distinction in their model of bladder tumorigenesis used in this article as a case study: decisive variables are those belonging to the equations of the three output phenotypes. Therefore, kali could allow non-decisive variables to not be matched.

Ultimately, this could allow the modeller to specify himself/herself what a physiological attractor is without having to consider a physiological and a pathological variant. This could also allow to no longer think in terms of physiological versus pathological attractors but just desirable ones. Moreover, implementing the second envisaged improvement could greatly facilitate the implementation of the first one because the goal would become to obtain desired attractors regardless if they are de novo or not.

## Supplementary Material

Appendix 1: recall of previous concepts

## Supplementary Material

Appendix 2: multivalued case

## Supplementary Material

Appendix 3: core of kali

## Supplementary Material

Appendix 4: case study equations

## Supplementary Material

Example network equations in text format

## Supplementary Material

Case study equations in text format

## Supplementary Material

The 1-therapeutic bullets of the case study

## Supplementary Material

The 2-therapeutic bullets of the case study.

## Data accessibility

The data have been uploaded as the electronic supplementary material.
