# Supplementary material for: Therapeutic target discovery using Boolean network attractors: improvements of kali
Source: R Soc Open Sci. 2018 Feb 14;5(2):171852. doi: 10.1098/rsos.171852 (PMC5830779; doi:10.1098/rsos.171852)
Supplement: Appendix 1: recall of previous concepts [file rsos171852supp1.pdf]

Therapeutic target discovery using Boolean network attractors: improvements of kali

Appendix 1: recall of previous concepts

Arnaud Poret, Carito Guziolowski

January 2, 2018

arnaud.poret@gmail.com (corresponding author)
carito.guziolowski@ls2n.fr
LS2N, UMR 6004
Nantes, France

Below are some important concepts introduced in the previous article [1] where the complete background was presented.

1 Biological networks

A network is a directed graph $G = (V, E)$ where $V = \{v_1, \ldots, v_n\}$ is the set containing the nodes of the network and $E = \{(v_{i,1}, v_{j,1}), \ldots, (v_{i,m}, v_{j,m})\}$ is the set containing the edges linking these nodes. In practice, nodes represent entities while edges represent binary relations $R \subset V^2$ involving them: $v_i \; R \; v_j$ [2]. It indicates that the node v_i exerts an influence on the node v_j. For example, in gene regulatory networks [3], v_i can be a transcription factor while v_j a gene product. The edges are frequently signed so that they indicate if v_i exerts a positive or a negative influence on v_j, such as an activation or an inhibition.

2 Boolean networks

A Boolean network is a network where nodes are Boolean variables x_i and edges (x_i, x_j) are the $is\ input\ of$ relation: $x_i\ is\ input\ of\ x_j$. Each variable x_i has $b_i \in [\![0, n]\!]$ inputs influencing its state. Note that $b_i = 0$ is possible. In this case, x_i is an input of the network. Depending on the updating scheme, at each iteration $k \in [\![k_0, k_{end}]\!]$, one or more x_i are updated using their associated Boolean transition function f_i. This function uses Boolean operators, typically $\wedge$ (*and*), $\vee$ (*or*) and $\neg$ (*not*), to specify how the inputs $x_{i,1}, \ldots, x_{i,b_i}$ of x_i have to be related to compute its value, as in the following pseudocode representing a synchronous updating:

for $k \leftarrow k_0, \ldots, k_{end}$
    $x_1 \leftarrow f_1(x_{1,1}, \ldots, x_{1,b_1})$
    $\vdots$
    $x_n \leftarrow f_n(x_{n,1}, \ldots, x_{n,b_n})$
end for

which can be written in a more concise form:

for $k \leftarrow k_0, \ldots, k_{end}$
    $\boldsymbol{x} \leftarrow \boldsymbol{f}(\boldsymbol{x})$
end for

where $\boldsymbol{f} = (f_1, \ldots, f_n)$ is the Boolean transition function of the network and $\boldsymbol{x} = (x_1, \ldots, x_n)$ is its state vector. The value of the state vector belongs to the state space $S = \{0, 1\}^n$, which is the set containing all the possible states of the network.

The set $A = \{a_1, \ldots, a_p\}$ containing the attractors of the network is its attractor set. An attractor a_i is a collection of states $(\boldsymbol{x}_1, \ldots, \boldsymbol{x}_q)$ such that once the system reaches a state $\boldsymbol{x}_j \in a_i$, it can subsequently visit the states of a_i but no other ones: the system can not escape. The set $B_i \subset S$ containing the states $\boldsymbol{x} \in S$ from which a_i can be reached is its basin of attraction, or simply basin.

3 Definitions

- **physiological phenotype**: a phenotype which does not impair the life quantity/quality of the organism which exhibits it
- **pathological phenotype**: a phenotype which impairs the life quantity/ quality of the organism which exhibits it
- **variant (of a biological network)**: given a biological network, a variant is one of its versions, namely the network plus eventually some modifications
- **physiological variant**: a variant which produces only physiological phenotypes, this is the biological network as it should be, the one of healthy organisms
- **pathological variant**: a variant which produces at least one pathological phenotype, this is a dysfunctional version of the biological network, a version found in ill organisms
- **physiological attractor set**: the attractor set A_{physio} of the physiological variant
- **pathological attractor set**: the attractor set A_{patho} of the pathological variant
- **physiological Boolean transition function**: the Boolean transition function $\boldsymbol{f}_{physio}$ of the physiological variant
- **pathological Boolean transition function**: the Boolean transition function $\boldsymbol{f}_{patho}$ of the pathological variant
- **physiological attractor**: an attractor a_i such that $a_i \in A_{physio}$, note that it does not exclude the possibility that $a_i \in A_{patho}$ in addition to $a_i \in A_{physio}$
- **pathological attractor**: an attractor a_i such that $a_i \notin A_{physio}$
- **modality**: the perturbation $moda_i \in \{0, 1\}$ applied on a node v_j of the network, either activating ($moda_i = 1$) or inactivating ($moda_i = 0$), at each iteration $moda_i$ overwrites $f_j(\boldsymbol{x})$ making $x_j = moda_i$
- **target**: a node $targ_i$ of the network on which a modality $moda_i$ is applied
- **bullet**: a couple (c_{targ}, c_{moda}) where $c_{targ} = (targ_1, \ldots, targ_r)$ is a combination without repetition of r targets and $c_{moda} = (moda_1, \ldots, moda_r)$ is an arrangement with repetition of r modalities, $moda_i$ is intended to be applied on $targ_i$

References

[1] Arnaud Poret and Jean-Pierre Boissel. An *in silico* target identification using boolean network attractors: avoiding pathological phenotypes. *Comptes Rendus Biologies*, 337(12):661–678, 2014.

[2] Xiaowei Zhu, Mark Gerstein, and Michael Snyder. Getting connected: analysis and principles of biological networks. *Genes & Development*, 21(9):1010–1024, 2007.

[3] Frank Emmert-Streib, Matthias Dehmer, and Benjamin Haibe-Kains. Gene regulatory networks and their applications: understanding biological and medical problems in terms of networks. *Frontiers in Cell and Developmental Biology*, 2:38, 2014.
