# Supplementary material for: Therapeutic target discovery using Boolean network attractors: improvements of kali
Source: R Soc Open Sci. 2018 Feb 14;5(2):171852. doi: 10.1098/rsos.171852 (PMC5830779; doi:10.1098/rsos.171852)
Supplement: Appendix 2: multivalued case [file rsos171852supp2.pdf]

Therapeutic target discovery using Boolean network attractors: improvements of kali

Appendix 2: multivalued case

Arnaud Poret, Carito Guziolowski

January 2, 2018

arnaud.poret@gmail.com (corresponding author)
carito.guziolowski@ls2n.fr
LS2N, UMR 6004
Nantes, France

Below is the multivalued version of the example network:

$$\begin{aligned}
do &= do \\
factory &= factory \\
energy &= max(min(energy, 1 - task), factory) \\
locker &= 1 - energy \\
releaser &= do \\
sequester &= 1 - releaser \\
activator &= min(do, 1 - locker) \\
effector &= min(activator, 1 - sequester) \\
task &= effector
\end{aligned}$$

where the Boolean operators are replaced by the Zadeh ones.

To take advantage of multivalued logic, f_{locker} becomes $locker = min(1 - energy, 0.5)$ in $\boldsymbol{f}_{patho}$. This equation tells that the locker is actionable when required, namely when there is no energy, but that it is unable at being fully operational due to some pathological defects: the maximal value of f_{locker} in $\boldsymbol{f}_{patho}$ is 0.5.

As mentioned in the article, 0.5 can be interpreted as an incomplete activation/inhibition depending on what is modeled. Consequently, in the pathological variant, the activator is at most partly inhibited by the locker when no energy is available, allowing the task to be nevertheless performed. However, in this case, the task is itself moderately performed.

1 Attractor sets

The example network is computed asynchronously using a 3-valued logic. As in the Boolean case, to compute an attractor set kali performs 1 000 random walks of 1 000 steps. Below are the returned attractors:

- A_{physio}:

attractor	basin (% of S_{physio})	*do*	*factory*	*energy*	*locker*	*task*
$a_{physio1}$	6.1%	0	0	0	1	0
$a_{physio2}$	4.5%	0	0	0.5	0.5	0
$a_{physio3}$	2.5%	0	0	1	0	0
$a_{physio4}$	9.7%	0	0.5	0.5	0.5	0
$a_{physio5}$	1.8%	0	0.5	1	0	0
$a_{physio6}$	10.8%	0	1	1	0	0
$a_{physio7}$	6.5%	0.5	0	0	1	0
$a_{physio8}$	4.8%	0.5	0	0.5	0.5	0.5
$a_{physio9}$	10.3%	0.5	0.5	0.5	0.5	0.5
$a_{physio10}$	10.6%	0.5	1	1	0	0.5
$a_{physio11}$	7.3%	1	0	0	1	0
$a_{physio12}$	3.2%	1	0	0.5	0.5	0.5
$a_{physio13}$	10.3%	1	0.5	0.5	0.5	0.5
$a_{physio14}$	11.6%	1	1	1	0	1

- A_{patho}:

attractor	basin (% of S_{patho})	*do*	*factory*	*energy*	*locker*	*task*
a_{patho1}	6.2%	0	0	0	0.5	0
$a_{physio2}$	4.7%	0	0	0.5	0.5	0
$a_{physio3}$	2.2%	0	0	1	0	0
$a_{physio4}$	9.7%	0	0.5	0.5	0.5	0
$a_{physio5}$	1.8%	0	0.5	1	0	0
$a_{physio6}$	10.8%	0	1	1	0	0
a_{patho2}	5.5%	0.5	0	0	0.5	0.5
$a_{physio8}$	5.8%	0.5	0	0.5	0.5	0.5
$a_{physio9}$	10.3%	0.5	0.5	0.5	0.5	0.5
$a_{physio10}$	10.6%	0.5	1	1	0	0.5
a_{patho3}	7.3%	1	0	0	0.5	0.5
$a_{physio12}$	3.2%	1	0	0.5	0.5	0.5
$a_{physio13}$	10.3%	1	0.5	0.5	0.5	0.5
$a_{physio14}$	11.6%	1	1	1	0	1

$a_{physio1}$, $a_{physio3}$, $a_{physio6}$, $a_{physio11}$ and $a_{physio14}$ are the physiological attractors found in the Boolean case with a different numbering due to additional attractors coming from multivalued logic. Given that $\{0, 1\} \subset \{0, 0.5, 1\}$ and that the Zadeh operators also work with Boolean logic, the Boolean results are still obtainable. The same does not apply to the pathological attractors because f_{locker} in $\boldsymbol{f}_{patho}$ differs between the Boolean and multivalued cases.

For example, $a_{physio13}$ indicates that the do instruction is sent while energy is partly supplied. Consequently, the locker is partly activated resulting in a partial inhibition of the activator. The task is thus moderately performed despite full do instruction, hence coping with moderate energy supply.

Concerning the pathological attractors, as an example a_{patho3} indicates that the do instruction is sent in absence of energy supply. Consequently, the locker should be fully activated to prevent the task. However, due to some pathological defects, the locker is at most partly activated. The task is then performed in absence of energy. However, since the locker is partly operational, the task is not performed at its maximum rate, maybe limiting pathological consequences.

Among the pathological attractors, a_{patho1} can be considered weakly pathological. Indeed, in a_{patho1} the locker should be fully activated since there is no energy. However, there is no do instruction and therefore no task to stop. On the other hand, a_{patho2} and a_{patho3} are more pathological since the task is performed while no energy is available.

2 Therapeutic bullets

All the bullets made of one to two targets are tested with a threshold of 5%. Below are the returned therapeutic bullets:

- 1-therapeutic bullets:

bullet	gain			$B_{physio1}$	$B_{physio2}$	$B_{physio3}$	$B_{physio4}$	$B_{physio5}$	$B_{physio6}$	$B_{physio7}$	$B_{physio8}$	$B_{physio9}$
				$B_{physio10}$	$B_{physio11}$	$B_{physio12}$	$B_{physio13}$	$B_{physio14}$	B_{patho1}	B_{patho2}	B_{patho3}	
$factory[0.5]$	81%	→	100%	0%	0%	0%	29.3%	6.1%	0%	0%	0%	32.2%
				0%	0%	0%	32.4%	0%	0%	0%	0%	
$factory[1]$	81%	→	100%	0%	0%	0%	0%	0%	35.4%	0%	0%	0%
				32.2%	0%	0%	0%	32.4%	0%	0%	0%	

- 2-therapeutic bullets:

bullet		gain			$B_{physio1}$	$B_{physio2}$	$B_{physio3}$	$B_{physio4}$	$B_{physio5}$	$B_{physio6}$	$B_{physio7}$	$B_{physio8}$	$B_{physio9}$
					$B_{physio10}$	$B_{physio11}$	$B_{physio12}$	$B_{physio13}$	$B_{physio14}$	B_{patho1}	B_{patho2}	B_{patho3}	
$do[0]$	$factory[0.5]$	81%	→	100%	0%	0%	0%	84%	16%	0%	0%	0%	0%
					0%	0%	0%	0%	0%	0%	0%	0%	
$do[0]$	$factory[1]$	81%	→	100%	0%	0%	0%	0%	0%	100%	0%	0%	0%
					0%	0%	0%	0%	0%	0%	0%	0%	
$do[0.5]$	$factory[0.5]$	81%	→	100%	0%	0%	0%	0%	0%	0%	0%	0%	100%
					0%	0%	0%	0%	0%	0%	0%	0%	
$do[0.5]$	$factory[1]$	81%	→	100%	0%	0%	0%	0%	0%	0%	0%	0%	0%
					100%	0%	0%	0%	0%	0%	0%	0%	
$do[1]$	$factory[0.5]$	81%	→	100%	0%	0%	0%	0%	0%	0%	0%	0%	0%
					0%	0%	0%	100%	0%	0%	0%	0%	
$do[1]$	$factory[1]$	81%	→	100%	0%	0%	0%	0%	0%	0%	0%	0%	0%
					0%	0%	0%	0%	100%	0%	0%	0%	
$do[0]$	$energy[1]$	81%	→	100%	0%	0%	34.9%	0%	32.1%	33%	0%	0%	0%
					0%	0%	0%	0%	0%	0%	0%	0%	
$do[0]$	$task[0]$	81%	→	89%	0%	11.6%	12.3%	21.3%	10.8%	33%	0%	0%	0%
					0%	0%	0%	0%	0%	11%	0%	0%	
$do[0.5]$	$task[0.5]$	81%	→	89.4%	0%	0%	0%	0%	0%	0%	0%	24.3%	32.1%
					33%	0%	0%	0%	0%	0%	10.6%	0%	
$factory[0]$	$energy[0.5]$	81%	→	100%	0%	35.4%	0%	0%	0%	0%	0%	32.2%	0%
					0%	0%	32.4%	0%	0%	0%	0%	0%	
$factory[0.5]$	$energy[0.5]$	81%	→	100%	0%	0%	0%	35.4%	0%	0%	0%	0%	32.2%
					0%	0%	0%	32.4%	0%	0%	0%	0%	
$factory[1]$	$energy[1]$	81%	→	100%	0%	0%	0%	0%	0%	35.4%	0%	0%	0%
					32.2%	0%	0%	0%	32.4%	0%	0%	0%	
$factory[1]$	$locker[0]$	81%	→	100%	0%	0%	0%	0%	0%	35.4%	0%	0%	0%
					32.2%	0%	0%	0%	32.4%	0%	0%	0%	

For example, the therapeutic bullet $factory[1]$ $locker[0]$ is interesting. It suppresses all the pathological attractors while maintaining three physiological attractors allowing the pathological variant to properly respond to the three possible levels of the do instruction. Moreover, the basins of these three physiological attractors, namely $a_{physio6}$, $a_{physio10}$ and $a_{physio14}$, equally span the state space, making them equally reachable.

On the other hand, the therapeutic bullet $do[0.5]$ $factory[0.5]$ seems to be less interesting. While this bullet also suppresses all the pathological attractors, it enables only one physiological attractor. In this physiological attractor, namely $a_{physio9}$, all the variables are at their intermediate level: the network can not fulfill its switching function.
