# Supplementary material for: Therapeutic target discovery using Boolean network attractors: improvements of kali
Source: R Soc Open Sci. 2018 Feb 14;5(2):171852. doi: 10.1098/rsos.171852 (PMC5830779; doi:10.1098/rsos.171852)
Supplement: Appendix 3: core of kali [file rsos171852supp3.pdf]

Therapeutic target discovery using Boolean network attractors: improvements of kali

Appendix 3: core of kali

Arnaud Poret, Carito Guziolowski

January 2, 2018

arnaud.poret@gmail.com (corresponding author)
carito.guziolowski@ls2n.fr
LS2N, UMR 6004
Nantes, France

Below is the core of kali in pseudocode derived from its Go [1] sources, freely available on GitHub at `https://github.com/arnaudporet/kali` under the GNU General Public License [2]. Note that the code may have evolved since the publication of the present article.

1 Defined types

structure *Attractor*// an attractor
 field *Name*// its name, either a_{physio} or a_{patho}
 field *Basin*// the size of its basin, in percents of the state space
 field *States*// its states, as a matrix of one state per row
end structure

structure *Bullet*// a bullet
 field *Targ*// its target combination, as a vector
 field *Moda*// its modality arrangement, as a vector
 field *Gain*// its gain, see below
 field *Cover*// the size of each basin under its influence, see below
end structure

$b.Gain$ is a vector $(gain_1, gain_2)$ where:

- $gain_1$ is the size of $\bigcup B_{physio,i}$ in S_{patho}
- $gain_2$ is the size of $\bigcup B_{physio,i}$ in S_{test}

in % of S_{patho} and % of S_{test} respectively.

$b.Cover$ is a vector containing the size of the physiological and pathological basins in the testing state space, in percents of it.

2 Parameters

nodes// the node names, as a vector
Ω// the domain of the used logic, as a vector
sync// use synchronous updating ($sync = 1$) or not ($sync = 0$)
whole// build the whole state space ($whole = 1$) or not ($whole = 0$)
max_S// the maximal size of the state space sample when $whole = 0$
max_k// the number of steps for the random walks (asynchronous only)
n_{targ}// the number of targets per bullet
max_{targ}// the maximum number of target combinations to test
max_{moda}// the maximum number of modality arrangements to test
δ// the threshold for a bullet to be therapeutic, in percents of the state space

When $whole = 0$, a subset of the state space is built. This subset contains the initial states from which trajectories are performed. These trajectories are used for computing an attractor set, that is to reach the attractors. Note that these trajectories are free to evolve in all the state space. In other words, kali does not run on a subset of the state space, these are the initial states which are a

subset of the state space.

To be considered therapeutic, a bullet has to make $gain_2 - gain_1 \geq \delta$ while not creating *de novo* attractors.

3 Functions

function $DoTheJob(f_{physio}, f_{patho}, n_{targ}, max_{targ}, max_{moda}, max_S, max_k, \delta,$
$sync, nodes, \Omega, whole)$
// do the job, this is the main function
$n \leftarrow Size(nodes)$// the dimension of the state space S
select $whole$
case 0// build a sample of S
$S \leftarrow GenArrangs(\Omega, n, max_S)$
case 1// build all S
$S \leftarrow GenSpace(\Omega, n)$
end select
$A_{physio} \leftarrow ComputeAttractorSet(f_{physio}, S, \varnothing, max_k, 0, sync, \emptyset)$
$A_{patho} \leftarrow ComputeAttractorSet(f_{patho}, S, \varnothing, max_k, 1, sync, A_{physio})$
$A_{versus} \leftarrow GetVersus(A_{patho})$// the pathological attractors, see below
$C_{targ} \leftarrow GenCombis(\{1, \ldots, n\}, n_{targ}, max_{targ})$// the target combinations
$C_{moda} \leftarrow GenArrangs(\Omega, n_{targ}, max_{moda})$// the modality arrangements
if $A_{versus} \neq \emptyset$// there are pathological basins to shrink
$B_{therap} \leftarrow ComputeTherapeuticBullets(f_{patho}, S, C_{targ}, C_{moda}, max_k,$
$\delta, sync, A_{physio}, A_{patho}, A_{versus})$// therapeutic bullets
end if
return $S, A_{physio}, A_{patho}, A_{versus}, C_{targ}, C_{moda}, B_{therap}$
end function

$Size(container)$ returns the number of items in $container$.

$GenSpace(\Omega, n)$ returns the n-dimensional state space of the vectors made of n values from Ω, as a matrix of one state vector per row.

$GenArrangs(\Omega, n, max_{arrang})$ returns max_{arrang} arrangements with repetition made of n elements from Ω, as a matrix of one arrangement per row. If max_{arrang} exceeds its maximal possible value then it is automatically decreased to its maximal possible value.

$GenCombis(\Omega, n, max_{combi})$ returns max_{combi} combinations without repetition made of n elements from Ω, as a matrix of one combination per row. If max_{combi} exceeds its maximal possible value then it is automatically decreased to its maximal possible value.

As explained later, the function $ComputeAttractorSet$ can use an already computed attractor set, namely the reference set, to name the attractors.

A_{physio} is computed without bullet ($b \leftarrow \varnothing$), without reference set ($A_{ref} \leftarrow \emptyset$)

and with the physiological setting ($setting \leftarrow 0$).

A_{patho} is computed without bullet ($b \leftarrow \varnothing$), with a reference set ($A_{ref} \leftarrow A_{physio}$) and with the pathological setting ($setting \leftarrow 1$).

A_{versus} is not a true attractor set but the set containing the pathological attractors: $A_{versus} \subset A_{patho}$. A_{patho} can contains physiological attractors if the pathological variant exhibits some of them. However, A_{versus} only contains the pathological attractors.

Therapeutic bullets are computed only if there are pathological basins to shrink, namely only if $A_{versus} \neq \emptyset$.

Note that target combinations are combinations of positions in the state vector: targets are identified by their position in the state vector, not by their name.

```
function f_physio(x)
    // update the state vector of the physiological variant
    y[1] ← f_physio[1](x)// update x_1 with f_physio,1
    ⋮
    y[n] ← f_physio[n](x)// update x_n with f_physio,n
    return y// the updated state vector
end function

function f_patho(x)
    // update the state vector of the pathological variant
    y[1] ← f_patho[1](x)// update x_1 with f_patho,1
    ⋮
    y[n] ← f_patho[n](x)// update x_n with f_patho,n
    return y// the updated state vector
end function

function ComputeAttractor(f, x_0, b, max_k, sync)
    // from x_0, reach an attractor a
    select sync
        case 1// search a cycle
            a.States ← ReachCycle(f, x_0, b)
        case 0// search a terminal SCC
            for
                a.States ← GoForward(f, Walk(f, x_0, b, max_k), b)// a candidate
                if IsTerminal(a, f, b)// the candidate attractor is a terminal SCC
                    break// then it is an asynchronous attractor
                end if
            end for
    end select
    return a
end function
```

If $ComputeAttractor(f, x_0, b, max_k, sync)$ is run with $sync = 0$ (i.e. asynchronous case) then ensure that max_k is big enough for random walks to reach attractors with high probability. If max_k is too small and if there is no attractor near x_0, then this function will run indefinitely since it loops until an attractor is found starting from x_0. The default value of max_k should be 10 000. It can be smaller for little networks and should be higher for large networks.

function $ComputeAttractorSet(f, S, b, max_k, setting, sync, A_{ref})$
    // compute an attractor set A, namely A_{physio}, A_{patho} or A_{test}
    $A \leftarrow \{\}$
    select $setting$// select the default name for attractors
        case 0// physiological setting
            $name \leftarrow a_{physio}$
        case 1// pathological setting
            $name \leftarrow a_{patho}$
    end select
    for $i \leftarrow 1, \ldots, Size(S)$// browse S
        $a \leftarrow ComputeAttractor(f, S[i], b, max_k, sync)$
        if $\exists i_A : A[i_A] = a$// a is already found
            $A[i_A].Basin \leftarrow A[i_A].Basin + 1$// then increase its basin
        else// new attractor
            $a.Basin \leftarrow 1$// then begin its basin
            $A \leftarrow A \cup \{a\}$// and add it to the attractor set
        end if
    end for
    for $i \leftarrow 1, \ldots, Size(A)$// browse A
        $A[i].Basin \leftarrow 100 \cdot A[i].Basin/Size(S)$// translate basins to % of S
    end for
    return $SetNames(A, name, A_{ref})$// return named attractors, see later
end function

function $ComputeTherapeuticBullets(f_{patho}, S, C_{targ}, C_{moda}, max_k, \delta, sync,$
    $A_{physio}, A_{patho}, A_{versus})$
    // compute a set B_{therap} of therapeutic bullets
    $B_{therap} \leftarrow \{\}$
    $b.Gain[1] \leftarrow Sum(GetCover(A_{physio}, A_{patho}))$// $\bigcup B_{physio,i}$ in S_{patho}
    for $i_1 \leftarrow 1, \ldots, Size(C_{targ})$// browse the target combinations to test
        for $i_2 \leftarrow 1, \ldots, Size(C_{Moda})$// browse the modality arrangements to test
            $b.Targ \leftarrow C_{targ}[i_1]$// the target combination to test
            $b.Moda \leftarrow C_{Moda}[i_2]$// the modality arrangement to test
            $A_{test} \leftarrow ComputeAttractorSet(f_{patho}, S, b, max_k, 1, sync, A_{physio})$
            $b.Gain[2] \leftarrow Sum(GetCover(A_{physio}, A_{test}))$// $\bigcup B_{physio,i}$ in S_{test}
            if $IsTherapeutic(b, A_{test}, A_{versus}, \delta)$// b is therapeutic
                $b.Cover \leftarrow GetCover(A_{physio} \cup A_{versus}, A_{test})$// basins in S_{test}
                $B_{therap} \leftarrow B_{therap} \cup \{b\}$// add b to the set of therapeutic bullets
            end if
        end for
    end for
    return B_{therap}
end function

$Sum(container)$ returns the sum of the items in $container$.

function $GetCover(A_1, A_2)$
    // get the size of the $B_{1,i}$ in S_2, in % of S_2
    $cover \leftarrow ()$
    for $i_1 \leftarrow 1, \ldots, Size(A_1)$// browse the attractors of A_1
        if $\exists i_2 : A_2[i_2] = A_1[i_1]$// $A_1[i_1]$ also in A_2
            $cover \leftarrow Append(cover, A_2[i_2].Basin)$// get the size of B_{1,i_1} in S_2
        else// $A_1[i_1]$ not in A_2
            $cover \leftarrow Append(cover, 0)$// then B_{1,i_1} is empty in S_2
        end if
    end for
    return $cover$
end function

$Append(container, item)$ returns $container$ with $item$ added to it.

function $GetVersus(A_{patho})$
    // get the pathological attractors
    $A_{versus} \leftarrow \{\}$// the set of the pathological attractors
    for $i \leftarrow 1, \ldots, Size(A_{patho})$// browse the attractors of A_{patho}
        if $IsSubString(A_{patho}[i].Name, patho)$// not a physiological attractor
            $A_{versus} \leftarrow A_{versus} \cup \{A_{patho}[i]\}$// then add it to A_{versus}
        end if
    end for
    return A_{versus}
end function

$IsSubString(s_1, s_2)$ returns $true$ if s_2 is a substring of s_1.

Remember that A_{versus} is not a true attractor set but the set containing the pathological attractors: $A_{versus} \subset A_{patho}$.

function $GoForward(f, x_0, b)$
    // compute the forward reachable set fwd of x_0 (asynchronous only)
    $fwd \leftarrow \{x_0\}$// fwd contains x_0 itself
    $stack \leftarrow (x_0)$// the stack of the states to check, see below
    for
        $x \leftarrow stack[Size(stack)]$// get the last stack element
        $stack \leftarrow stack[1, \ldots, Size(stack) - 1]$// remove the last stack element
        $y \leftarrow f(x)$// prepare all the updated x_i
        for $i \leftarrow 1, \ldots, Size(y)$// browse the updated x_i
            $z \leftarrow x$// copy x to preserve its original value
            $z[i] \leftarrow y[i]$// update only one x_i
            $z \leftarrow Shoot(z, b)$// apply the bullet
            if $z \notin fwd$// new state
                $fwd \leftarrow fwd \cup \{z\}$// then add it to fwd
                $stack \leftarrow Append(stack, z)$// and add it to the states to check
            end if

```
        end for
        if Size(stack) = 0// no new states to visit
            break// so the complete fwd is obtained
        end if
    end for
    return fwd
end function
```

stack is the stack of the visited states for which the possible successors are not yet computed. Once this stack empty, all the visitable states starting from x_0 are found, that is the complete forward reachable set of x_0 is obtained.

```
function IsTerminal(a, f, b)
    // check if a candidate attractor a is a terminal SCC (asynchronous only)
    for i ← 1, ..., Size(a.States)// browse the states of a
        if GoForward(f, a.States[i], b) ≠ a.States// fwd_i ≠ a
            return false// then not a terminal SCC
        end if
    end for
    return true// assumed to be a terminal SCC until proven otherwise
end function

function IsTherapeutic(b, A_test, A_versus, δ)
    // check if a bullet b is therapeutic
    if b.Gain[2] − b.Gain[1] ≥ δ// maybe therapeutic
        for i ← 1, ..., Size(A_test)// browse the attractors of A_test
            if IsSubString(A_test[i].Name, patho) ∧ A_test[i] ∉ A_versus
                return false// because of a de novo attractor
            end if
        end for
        return true// assumed to be therapeutic until proven otherwise
    else
        return false// below the therapeutic threshold
    end if
end function

function ReachCycle(f, x_0, b)
    // compute the cycle reachable from x_0 (synchronous only)
    cycle ← (x_0)// begin the trajectory
    x ← x_0
    for
        x ← Shoot(f(x), b)// update and apply the bullet
        if ∃i : cycle[i] = x// cycle found in the trajectory
            cycle ← cycle[i, ..., Size(cycle)]// then extract the cycle
            break// mission completed
        else// cycle not yet reached
            cycle ← Append(cycle, x)// then pursue the trajectory
        end if
    end for
    return cycle
```

```
end function

function SetNames(A, name, A_ref)
    // name the attractors of A according to A_ref, default to name
    y ← A// copy A to return a copy
    k ← 1// initiate the default name numbering
    for i ← 1, ..., Size(A)// browse the attractors of A
        if ∃i_ref : A_ref[i_ref] = A[i]// A[i] found in A_ref
            y[i].Name ← A_ref[i_ref].Name// then get its name in A_ref
        else// A[i] not in A_ref
            y[i].Name ← CatStrings(name, ToString(k))// default name
            k ← k + 1// and increment the default name numbering
        end if
    end for
    return y
end function
```

$CatStrings(s_1, s_2)$ returns the concatenation of s_1 and s_2.

$ToString(item)$ returns the string corresponding to $item$.

This function names the attractors of A according to a reference set A_{ref}. If an attractor of A also belongs to A_{ref} then its name in A_{ref} is used, otherwise the default name numbered with k is used.

```
function Shoot(x, b)
    // apply a bullet on a state
    y ← x// copy x to return a copy
    for i ← 1, ..., Size(b.Targ)// browse the targets
        y[b.Targ[i]] ← b.Moda[i]// apply the corresponding modality
    end for
    return y
end function
```

Remember that targets are identified by their position in the state vector, not by their name.

```
function Walk(f, x_0, b, max_k)
    // perform a random walk of max_k steps from x_0 (asynchronous only)
    x ← x_0// start the walk
    for k ← 1, ..., max_k// for max_k steps
        y ← f(x)// prepare all the updated x_i
        i ← RandInt(1, Size(x))// randomly choose one x_i
        x[i] ← y[i]// then update the chosen x_i
        x ← Shoot(x, b)// and apply the bullet
    end for
    return x
end function
```

$RandInt(a, b)$ returns a randomly selected integer in $[\![a; b]\!]$ according to a uni-

form distribution.

References

[1] The go programming language. `https://golang.org`.

[2] The gnu general public license. `https://www.gnu.org/licenses/gpl.html`.
