# Supplementary material for: Therapeutic target discovery using Boolean network attractors: improvements of kali
Source: R Soc Open Sci. 2018 Feb 14;5(2):171852. doi: 10.1098/rsos.171852 (PMC5830779; doi:10.1098/rsos.171852)
Supplement: Appendix 4: case study equations [file rsos171852supp4.pdf]

Therapeutic target discovery using Boolean network attractors: improvements of kali

Appendix 4: case study equations

Arnaud Poret, Carito Guziolowski

January 2, 2018

arnaud.poret@gmail.com (corresponding author)
carito.guziolowski@ls2n.fr
LS2N, UMR 6004
Nantes, France

Below are the 27 Boolean equations of the case study derived from the model of bladder tumorigenesis by Elisabeth Remy and colleagues [1]. These equations are also available in text format in the supporting file `bladder_equations.txt`. A network-based representation is shown in the `Figure 2` of the article.

$$
\begin{aligned}
AKT &= PI3K \\
ATM_{lvl1} &= DNAdamage \land \neg E2F1_{lvl1} \land \neg E2F1_{lvl2} \\
ATM_{lvl2} &= (E2F1_{lvl1} \lor E2F1_{lvl2}) \land DNAdamage \\
CDC25A &= \neg CHEK1/2_{lvl1} \land \neg CHEK1/2_{lvl2} \land \neg RBL2 \\
&\quad \land (E2F1_{lvl1} \lor E2F1_{lvl2} \lor E2F3_{lvl1} \lor E2F3_{lvl2}) \\
CHEK1/2_{lvl1} &= (ATM_{lvl1} \lor ATM_{lvl2}) \land \neg E2F1_{lvl1} \land \neg E2F1_{lvl2} \\
CHEK1/2_{lvl2} &= (E2F1_{lvl1} \lor E2F1_{lvl2}) \land (ATM_{lvl1} \lor ATM_{lvl2}) \\
CyclinA &= \neg RBL2 \land \neg p21CIP \land CDC25A \\
&\quad \land (E2F1_{lvl1} \lor E2F1_{lvl2} \lor E2F3_{lvl1} \lor E2F3_{lvl2}) \\
CyclinD1 &= (RAS \lor AKT) \land \neg p16INK4a \land \neg p21CIP \\
CyclinE1 &= \neg RBL2 \land \neg p21CIP \land CDC25A \\
&\quad \land (E2F1_{lvl1} \lor E2F1_{lvl2} \lor E2F3_{lvl1} \lor E2F3_{lvl2}) \\
E2F1_{lvl1} &= \neg RB1 \land \neg RBL2 \land ((CHEK1/2_{lvl2} \land ATM_{lvl2} \land \neg RAS \land E2F3_{lvl1}) \\
&\quad \lor ((\neg CHEK1/2_{lvl2} \lor \neg ATM_{lvl2}) \land (RAS \lor E2F3_{lvl1} \lor E2F3_{lvl2}))) \\
E2F1_{lvl2} &= \neg RBL2 \land \neg RB1 \land ATM_{lvl2} \land CHEK1/2_{lvl2} \land (RAS \lor E2F3_{lvl2}) \\
E2F3_{lvl1} &= \neg RB1 \land \neg CHEK1/2_{lvl2} \land RAS \\
E2F3_{lvl2} &= \neg RB1 \land CHEK1/2_{lvl2} \land RAS \\
EGFR &= (EGFRstimulus \lor SPRY) \land \neg FGFR3 \land \neg GRB2 \\
FGFR3 &= \neg EGFR \land FGFR3stimulus \land \neg GRB2 \\
GRB2 &= (FGFR3 \land \neg GRB2 \land \neg SPRY) \lor EGFR \\
MDM2 &= (TP53 \lor AKT) \land \neg p14ARF \land \neg ATM_{lvl1} \land \neg ATM_{lvl2} \land \neg RB1 \\
p14ARF &= E2F1_{lvl1} \lor E2F1_{lvl2} \\
p16INK4a &= GrowthInhibitors \land \neg RB1 \\
p21CIP &= \neg CyclinE1 \land (GrowthInhibitors \lor TP53) \land \neg AKT \\
PI3K &= GRB2 \land RAS \land \neg PTEN \\
PTEN &= TP53 \\
RAS &= EGFR \lor FGFR3 \lor GRB2 \\
RB1 &= \neg CyclinD1 \land \neg CyclinE1 \land \neg p16INK4a \land \neg CyclinA \\
RBL2 &= \neg CyclinD1 \land \neg CyclinE1 \\
SPRY &= RAS \\
TP53 &= \neg MDM2 \land (E2F1_{lvl2} \lor ((ATM_{lvl1} \lor ATM_{lvl2}) \\
&\quad \land (CHEK1/2_{lvl1} \lor CHEK1/2_{lvl2})))
\end{aligned}
$$

The four input parameters are $EGFRstimulus$, $FGFR3stimulus$, $GrowthInhibitors$ and $DNAdamage$. The three outputs are evaluated from the returned attractors once the run terminated according to their respective equation:

$$
\begin{aligned}
Proliferation &= CyclinE1 \lor CyclinA \\
GrowthArrest &= p21CIP \lor RB1 \lor RBL2 \\
Apoptosis &= TP53 \lor E2F1_{lvl2}
\end{aligned}
$$

References

[1] Elisabeth Remy, Sandra Rebouissou, Claudine Chaouiya, Andrei Zinovyev, Francois Radvanyi, and Laurence Calzone. A modeling approach to explain mutually exclusive and co-occurring genetic alterations in bladder tumorigenesis. *Cancer Research*, 75(19):4042–4052, 2015.
